# Differential Immune Checkpoint Protein Expression in HNSCC: The Role of HGF/MET Signaling

**DOI:** 10.3390/ijms25137334

**Published:** 2024-07-04

**Authors:** Verena Boschert, Johannes Boenke, Ann-Kathrin Böhm, Jonas Teusch, Valentin Steinacker, Anton Straub, Stefan Hartmann

**Affiliations:** Department of Oral and Maxillofacial Plastic Surgery, University Hospital Wuerzburg, D-97070 Würzburg, Germany

**Keywords:** HNSCC, MET, HGF, PD-L2, PD-L1, VISTA, B7-H4, ICI, ICOSL, EMT

## Abstract

Although inhibitors targeting the PD1/PD-L1 immune checkpoint are showing comparably good outcomes, a significant percentage of head and neck squamous cell carcinoma (HNSCC) patients do not respond to treatment. Apart from using different treatment strategies, another possibility would be to target other immune checkpoints operating in these non-responding tumors. To obtain an overview of which checkpoint ligands are expressed on HNSCC tumor cells and if these ligands are affected by HGF/MET signaling, we used mRNA sequencing and antibody-based techniques for identifying checkpoint ligands in six HNSCC tumor cell lines. Furthermore, we compared our results to mRNA sequencing data. From the checkpoint ligands we investigated, VISTA was expressed the highest at the RNA level and was also the most ubiquitously expressed. PD-L2 and B7-H3 were expressed comparably lower and were not present in all cell lines to the same extent. B7-H4, however, was only detectable in the Detroit 562 cell line. Concerning the effect of HGF on the ligand levels, PD-L2 expression was enhanced with HGF stimulation, whereas other checkpoint ligand levels decreased with stimulation. B7-H4 levels in the Detroit 562 cell line drastically decreased with HGF stimulation. This is of interest because both the checkpoint ligand and the growth factor are reported to be connected to epithelial–mesenchymal transition in the literature.

## 1. Introduction

Immune checkpoint inhibitors (ICIs) were the big breakthrough in cancer treatment in recent years. The development of these inhibitors is based upon the fact that tumors can evade the immune system by expressing components of different control systems that enable T cells to discriminate between healthy and degenerated cells. Taking the PD-1/PD-L1 checkpoint as an example, upon the interaction of a ligand on a tumor cell (PD-L1, programmed death ligand 1) and a checkpoint receptor (PD-1, programmed cell death protein) on a T cell, activation of the immune cell is prevented. Cancer cells can achieve the expression of checkpoint ligands by cell intrinsic mechanisms via oncogenic events. In addition, inflammatory cytokines, like interferon-γ produced by initially activated T cells in the tumor microenvironment, enhance the expression of immune checkpoint ligands. Not only is their expression in tumor cells induced, but also in other antigen-representing cell types of the tumor microenvironment, such as macrophages, dendritic and stromal cells [1]. Altogether, this leads to the suppression of tumor-specific responses of the T cells and subsequently to immune evasion. Using specific inhibitors to impede the interaction of checkpoint receptors and ligands enables the immune system to control tumor cells, and the patient being treated could benefit from that.

Inhibitors acting on the PD1/PD-L1 immune checkpoint have been very successful in a range of tumor entities, e.g., in resected stage III/IV melanoma [2] or non-small cell lung cancer [3]. For head and neck squamous cell carcinoma (HNSCC), the PD-1 specific ICI Pembrolizumab was approved by the U.S. Food and Drug Administration and the European Commission for treatment of recurrent and metastatic tumors in 2019 either as a monotherapy or in combination with platinum and 5-fluorouracil (5-FU) chemotherapy. There was a significantly higher survival benefit (overall survival of 13.6 vs. 10.4 months) in PD-L1-positive participants treated with Pembrolizumab in combination with chemotherapy compared to the standard treatment (Cetuximab in combination with chemotherapy) in the corresponding KEYNOTE-048 trial (NCT02358031 [4]). However, an overall response rate (ORR) of only 36.4% was achieved for this combination treatment in PDL1-positive patients. Blocking PD1/PD-L1 signaling with a single agent (without chemotherapy) typically results in a response rate between 15% and 20%. The reasons for this low response rates have been discussed and investigated [5,6]. Strategies to target different immune checkpoints or to use combination treatments together with reagents targeting other signaling pathways to improve treatment of the disease are currently being investigated.

Most HNSCCs are derived from the mucosal squamous epithelial cells of different locations of the upper aero digestive tract. Excessive tobacco and alcohol consumption are the most common risk factors for the disease, but especially in the oropharynx, cases are also very often linked to former human papillomavirus (HPV) infections [7]. Several growth factor signaling pathways in the HNSCC tumor cells have been shown to contribute to cancer progression. Comparable to other epithelial cancer forms, the EGF/EGFR (epidermal growth factor/epidermal growth factor receptor) signaling pathway was found to be abnormally activated in a majority of HNSCC tumors [8,9]. Therefore, treatment with the EGFR-specific antibody Cetuximab is an available targeted therapy for HNSCC. In combination with radiotherapy, it has shown a significantly higher overall survival rate (62% vs. 55% after two years) together with a higher response rate (74% vs. 64%) compared to radiotherapy treatment alone [10]. Unfortunately, many patients show an initial or acquired resistance to this treatment.

Another pathway of relevance for HNSCC is the HGF/MET (hepatocyte growth factor/mesenchymal epithelial transition factor) pathway. High MET expression is associated with worse survival outcomes and higher disease stages [11,12,13]. Only very few treatments in the clinic are targeting HGF/MET signaling up to today. Although there were several trials trying to establish a MET-specific therapy for different cancer entities, only for cases of NSCLC with a specific mutation in MET (exon 14 skipping) are tyrosine kinase inhibitors targeting MET approved for therapy (Tepotinib and Capmatinib) [14]. For Tepotinib, one trial eligible for HNSCC patients is currently recruiting participants (NCT04647838). With antibody-based agents targeting HGF/MET signaling [15], two trails for HNSCC are currently ongoing, one with an HGF-specific antibody (Ficlatuzumab, Phase III, NCT06064877) and the second with an MET-EGFR-bispecific antibody (Phase I/II, MCLA-129, NCT04868877).

The HGF/MET pathway impacts tumorigenesis of HNSCC by mutations, overexpression or amplification of the MET or HGF genes. Furthermore, it can drive metastasis by promoting epithelial–mesenchymal transition [16]. An active HGF/MET pathway enhances HNSCC tumor cell proliferation by increasing glycolysis [17]. It was also found to be one of the players conferring resistance to Cetuximab treatment [18]. Furthermore, it can influence immune evasion by increasing expression of the immune checkpoint ligand PD-L1 on HNSCC tumor cells [19].

In regard for the need for an optimized treatment with ICIs with higher response rates in HNSCC, we were interested if HGF stimulation could also have an impact on the expression of other immune checkpoint ligands apart from PD-L1. Therefore, we looked at the expression of a set of immune checkpoint ligands in different HNSCC cell lines under HGF stimulation or additional MET inhibition in comparison to the respective untreated condition. We also analyzed public mRNA expression data sets of a large range of tumors of different entities to set our results into a broader context.

## 2. Results

**Figure 1:**
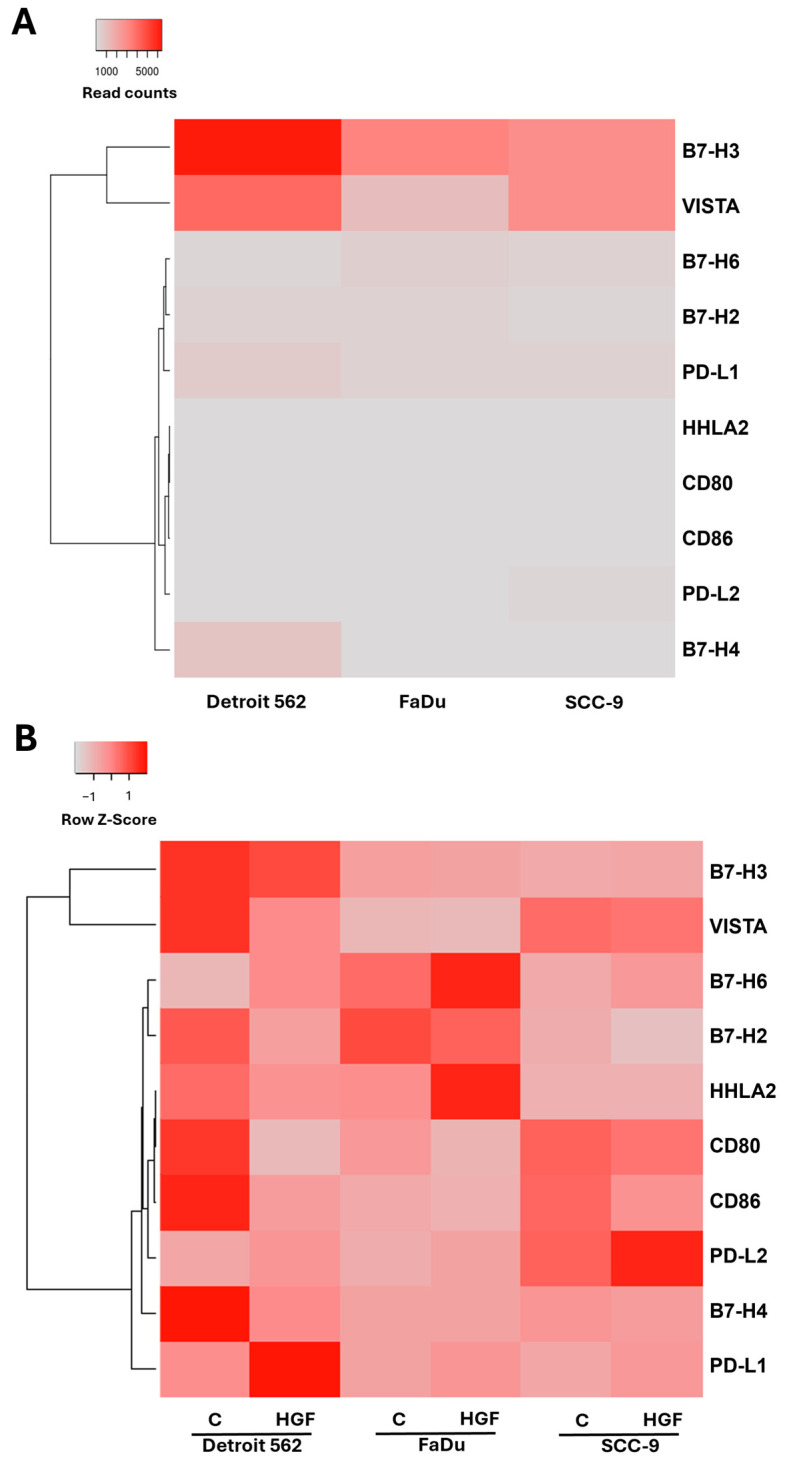
mRNA expression of genes in B7 protein family in three HNSCC cell lines. Results of mRNA sequencing of cell lines Detroit 562, FaDu and SCC-9 for all B7 protein family members. (**A**) Absolute expression (normalized read counts) in all three lines in untreated control sample. (**B**) Relative expression for each gene in control (C) and HGF-treated (HGF) samples (row z-score scaling).

To investigate the effects of HGF stimulation on immune checkpoint ligands in HNSCC, we analyzed mRNA sequencing data from three cell lines. The HNSCC cell lines Detroit 562, SCC9 and FaDu were stimulated with 50 ng/mL HGF for 16 h or were left untreated and subjected to RNA isolation and mRNA sequencing. The original data we already published in 2020 [17]; we now reassessed the data with focus on a list of common checkpoint proteins (Figure 1).

Comparing the normalized read counts between the untreated cell lines (Figure 1A), one can see that B7-H3 and VISTA are expressed the highest in all three lines, whereas the other checkpoint proteins are expressed comparably lower. When we look at the relative z-score values for each gene and compare untreated cells with HGF-stimulated cells, the effects of the treatment on the expression of the B7 protein family become visible (Figure 1B). As we already determined in an earlier study, PD-L1 expression is enhanced upon HGF stimulation in all three cell lines [19]. For PD-L2, the other known PD-1 ligand, this seems also to be the case, as the z-score in the treated samples is higher than in the control (Figure 1B). In contrast, for most of the other genes, expression is downregulated upon HGF stimulation.

**Figure 2:**
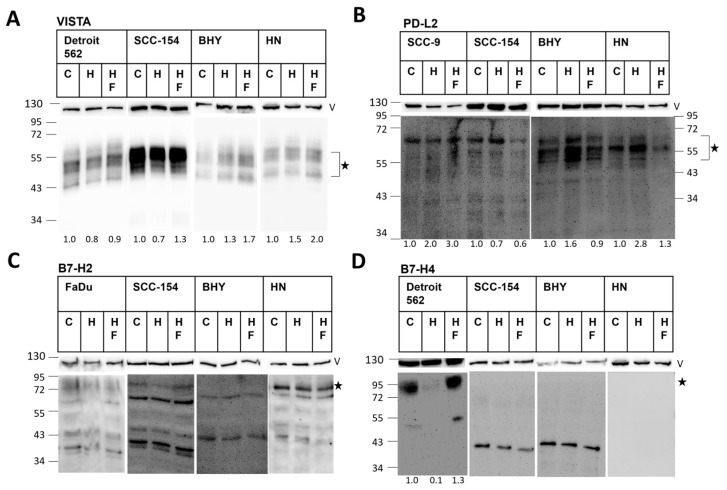
Protein expression of four selected B7 family members in different HNSCC cell lines. Shown are results of Western blots using protein samples isolated from the indicated cell lines stimulated for 6 h (**B**) or 24 h (**A**,**C**,**D**) with 50 ng/mL HGF (H), 50 ng/mL HGF and 0.5 µM Foretinib (HF) or left untreated (**C**). Blots were incubated with antibodies specific for VISTA (**A**), PD-L2 (**B**), B7-H2 (**C**) and B7-H4 (**D**). The upper parts of the blots were incubated with an antibody specific for Vinculin (V), and these signals served as loading controls. Molecular weights are indicated on the left and right in kDa. Asterisks indicate the expected height of the specific signal. The numbers beneath the blots show results of quantification, normalized to the untreated control. In (**C**,**D**) (except for Detroit 562) quantification was not possible because of no or too many unspecific signals.

**Figure 3:**
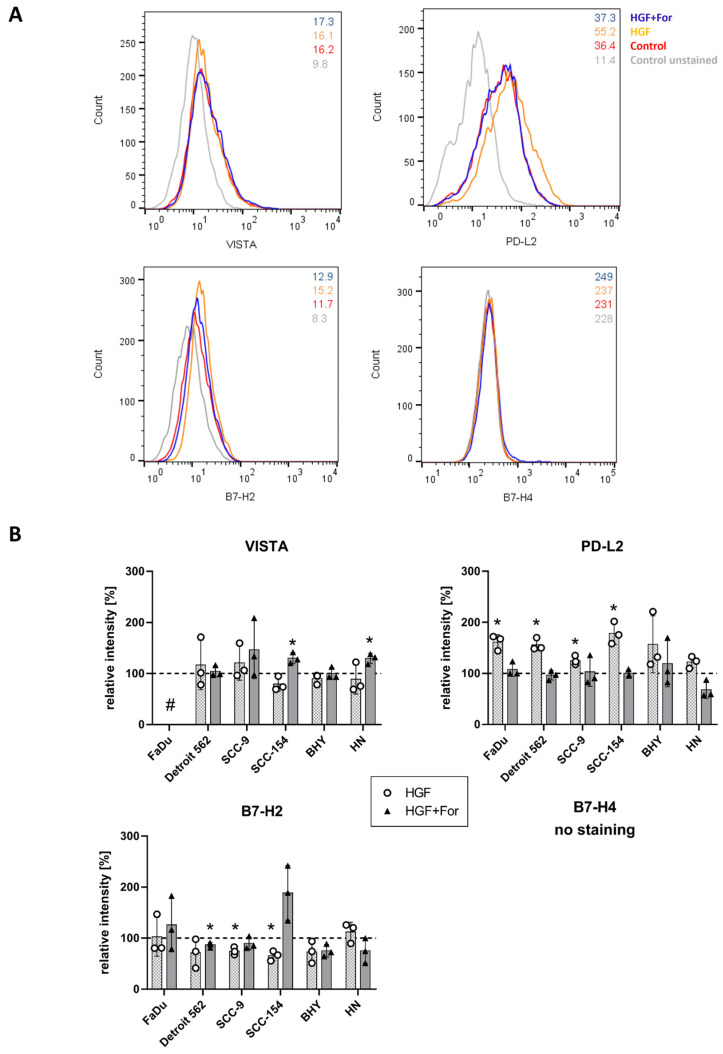
Expression of four different B7 family members on the cell surface of HNSCC cell lines. (**A**) Detroit 562 cells were stained with fluorophore-coupled antibodies specifically recognizing VISTA, PD-L2, B7-H2 and B7-H4 and subjected to flow cytometry. Histograms show results of HGF-treated cells (orange), those treated with HGF and Foretinib (blue) or untreated controls (red). Numbers indicate the median of the fluorescence intensity of the corresponding sample. Unstained sample of untreated control is shown for comparison. Relative fluorescence intensity on the *x* axis is plotted against cell count. (**B**) Overview of flow cytometry results in all six cell lines. Median values of fluorescence intensity were determined, and median values of corresponding unstained samples were subtracted. Mean values of the median fluorescence intensity of at least three independent experiments were plotted in relation to the untreated control (dashed line). For protein B7-H4, no staining could be determined on the cell surface of all cell lines investigated. The data of this figure can be found in the supplementary materials Table S1. #: no staining, *: *p*-value < 0.05 in a one-sample *t*-test.

To investigate if these effects are also visible at the protein level, we performed a Western blot (Figure 2) and flow cytometry (Figure 3) for a set of checkpoint proteins with our three cell lines. Additionally, we included three other HNSCC cell lines (HN, SCC154 and BHY) to obtain a broader picture of the effects of HGF stimulation in HNSCCs. To be able to check for MET specificity of the observable effects, we additionally applied the MET-specific tyrosine kinase inhibitor Foretinib. VISTA could be detected easily, showing a strong signal in all cell lines (Figure 2A, additional results in Figure S1A). This corresponds to the strong signal obtained for VISTA in the sequencing data (Figure 1A). In some cases, a drop in protein concentration was found upon HGF stimulation. The amount of PD-L2, on the other hand, clearly increased with HGF stimulation in most of the lines, but did not increase when Foretinib was added (Figure 2B and Figure S1B). B7-H2 was very difficult to detect. When there were signals of the correct protein size, there were too many additional, unspecific bands (Figure 2C and Figure S1C). No clear conclusions can therefore be drawn from these results on the effect of HGF on the protein level for B7-H2. B7-H4 was only detected effectively in Detroit 562 cells, and the protein amount was clearly reduced upon HGF stimulation (Figure 2D, additional results in Figure S1D).

Using flow cytometry, we wanted to investigate the occurrence of the immune checkpoint proteins on the cell surface; hence, we did not permeabilize cells before staining. As an example, staining of the Detroit 562 cell line is shown in Figure 3A. VISTA (top left) and PD-L2 (top right) could be clearly detected on the cell surface. For PD-L2, a clear shift towards higher staining can be seen when cells were treated with HGF. When inhibiting MET with the MET inhibitor Foretinib, this shift was absent. VISTA staining was not affected by either treatment. B7H2 (bottom left) showed only a weak staining. The median fluorescence intensity was slightly higher (15.2 versus 11.7 of the control) when HGF was added and became lower again when Foretinib was added (12.9 versus 11.7 of the control). For B7-H4, no surface staining could be detected at all (bottom right). Figure 3B shows a quantification of all flow cytometry results with all cell lines and proteins. Whereas in SCC154 and BHY cells less VISTA protein could be detected on the surface upon HGF stimulation, indicated by a lower median fluorescence intensity compared to the control, this difference was not statistically significant. In FaDu cells, the staining for VISTA was too weak to be analyzed. PD-L2 protein levels increased on the cell surface in all cells upon HGF stimulation. This could be prevented by adding Foretinib, showing the MET specificity of the effect of HGF stimulation. B7-H2 abundance on the cell surface was reduced in some cell lines upon HGF stimulation, but only in SCC9 and SCC154 cells did this effect reach statistical significance. Lastly, B7-H4 could not be detected in any of the cell lines using flow cytometry.

As B7-H4 has been already reported to be not easily detectable in flow cytometry [20,21], we additionally tried to detect the protein using immunofluorescence on fixed and permeabilized cells. The Western blot and sequencing results showed that only Detroit 562 is expressing the protein in larger amounts, so we decided to have a look only at this cell line while using the HN cell line as a low-expressing control. Additionally, we stained for PD-L1 to see if we could confirm our previous finding of enhanced PD-L1 expression upon HGF stimulation in HNSCC [19] with this method as well. Nuclei of cells were stained with Hoechst 33342 to be able to identify the cells and perform quantification of the results.

**Figure 4:**
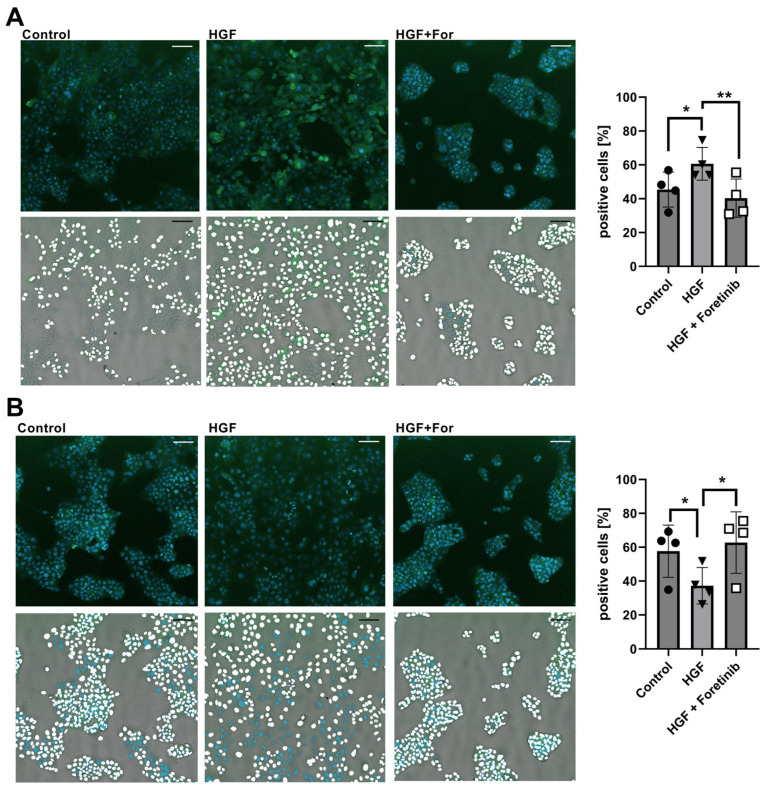
Immunofluorescent staining for PD-L1 and B7-H4 of Detroit 562 cells. Shown are results of a cell-scoring analysis using nuclear staining with Hoechst 33342 for cell identification and Alexa Fluor 488-coupled antibodies for staining for PD-L1 (**A**) or B7-H4 (**B**). Cells were fixed and permeabilized to simultaneously investigate membrane and intracellular staining. Before fixation, cells were kept untreated (left panel), treated with HGF (middle panel) or treated with HGF and Foretinib (right panel) for 24 h. Upper row of pictures shows overlays of the Hoechst 33342 (cyan) and Alexa Fluor 488 channels (green); lower row additionally depicts the transmitted light channels of the same pictures, and the results of the cell-scoring analysis are depicted as such, that those that scored positive for PD-L1 or B7-H4 are indicated by a white-stained nucleus. Diagrams on the right are showing the mean percentage of cells identified as positive for PD-L1 or B7-H4 from 4 independent experiments. Circles: control, inverted triangle: HGF treated, square: HGF and Foretinib treated. Shown are representative sections of total images. Ten pictures per condition were analyzed for each experiment. Bars correspond to 91.25 µm. *: *p* < 0.0332, **: *p* < 0.0021 in an unpaired *t*-test.

In Detroit 562 cells, we could detect a specific staining for PD-L1 in the cytoplasm and a distinct staining of the cell membrane (Figure 4A left panel). Upon HGF treatment, cells showed a scattering effect and a higher amount of cells typical for HGF (Figure 4A middle panel), which was not visible with Foretinib (Figure 4A right panel). A clear increase in staining intensity (upper panels) and a higher score of positive cells (lower panels) was visible in the HGF-treated sample (Figure 4A middle panel). This increase was prevented by adding Foretinib in addition to HGF (Figure 4A right panel). The quantification of four independent experiments indeed showed that HGF-treated samples contained a significantly higher fraction of PD-L1-positive cells compared to the untreated control and the samples additionally treated with Foretinib (Figure 4A diagram). On the other hand, B7-H4 could be specifically detected in Detroit 562 cells in a cytoplasmatic staining pattern without a distinct membrane staining (Figure 4B left panel). With HGF treatment (Figure 4B middle panel), this staining was weaker (upper panel), and fewer positive cells were scored (lower panel), whereas additional treatment with Foretinib resulted in a staining intensity and a number of positive cells comparable to the untreated control (Figure 4B right panel). The quantification of four independent experiments confirmed this effect: Significantly lower percentages of B7-H4-positive cells in the HGF-treated compared to the additionally Foretinib-treated as well as the untreated control group were found (Figure 4B diagram). In the HN cell line, for PD-L1 staining, the percentage of positive cells was also higher in HGF-treated cells, but the effect was not as pronounced (Figure S2A). The difference between HGF-treated samples with and without Foretinib-treated samples was significant. Comparable to the Western blot and sequencing results, the staining for B7-H4 was very weak in HN cells, and quantification revealed no significant differences between the sample groups (Figure S2B).

In summary, among the investigated checkpoint proteins, VISTA was expressed at the highest level and detected in nearly all investigated lines, whereas PD-L2 and B7-H2 were expressed consistently less. B7-H4 was only found at the protein level in one cell line out of six. Concerning the reaction upon HGF stimulation, we could confirm the mRNAseq data using Western blot and flow cytometry. Similar to the closely related PD-L1 protein, PD-L2 increases upon stimulation, and the other proteins we investigated rather decrease. B7-H4 could not be detected on the cell surface, but using immunofluorescence on permeabilized cells, its decrease upon HGF stimulation could be confirmed as well.

**Figure 5:**
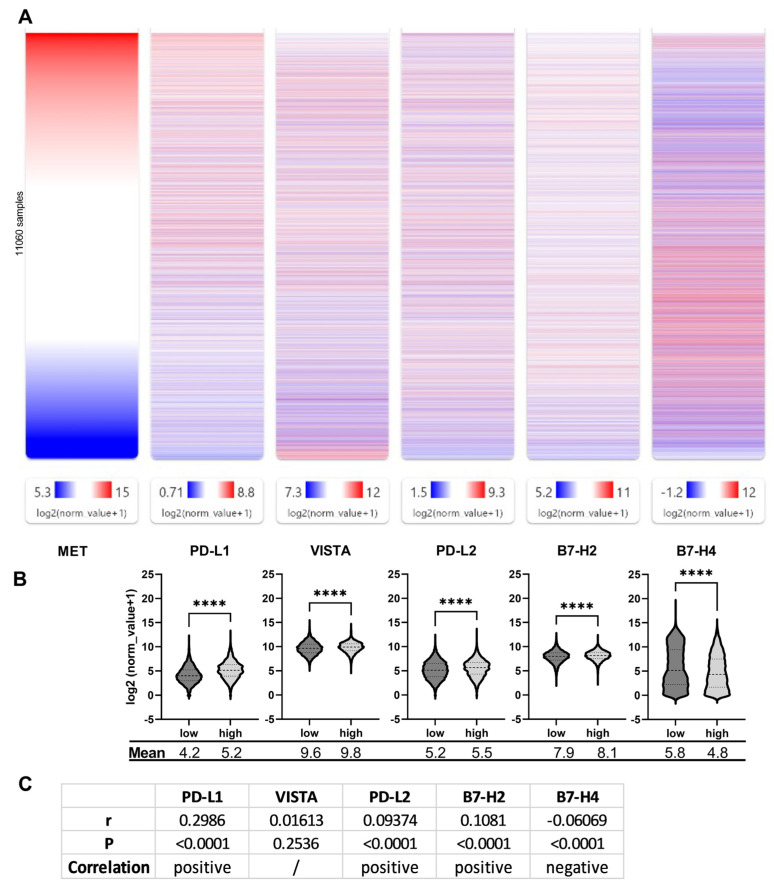
Correlation of MET and immune checkpoint protein expression. (**A**) mRNAseq data of immune checkpoint proteins PD-L1, VISTA, PD-L2, B7-H2 und B7-H4 was aligned to the mRNAseq data of MET with UCSC Xena using the TCGA PAN-Cancer (PANCAN) data set (sample type Solid Tissue Normal excluded). (**B**) TCGA PAN-Cancer data set (without Solid Tissue Normal) was downloaded from UCSC XENA and expression values for indicated proteins were grouped in samples with a MET expression lower (low) or higher (high) than the median MET expression value of 10.7. The resulting mean values of the two groups are indicated at the bottom of the graph. ****: *p* < 0.0001 in a t-test with Welch’s correction. (**C**) Spearman analysis of correlation was performed with the ungrouped data of (**B**) using GraphPad Prism. Indicated are the Spearman rho (r), the corresponding *p*-value (*p*) and the type of correlation. /: no correlation, α = 0.05.

To set these results in correlation with more physiological data obtained directly from tumors, we analyzed published mRNA sequencing data for the correlation of MET expression and checkpoint protein expression. Therefore, we loaded the TCGA PAN-Cancer data set in UCSC Xena and aligned the expression results of MET from the lowest- (dark blue) to the highest-expressing (dark red) samples with the results for PD-L1, VISTA, PD-L2, B7-H3 and B7-H4 (Figure 5A). The resulting heat maps for PD-L1 indicate that tumor samples with high PD-L1 expression showed high MET expression as well. Lower MET expression coincided with lower PD-L1 expression. For VISTA, PD-L2 and B7-H3, a clear pattern can be spotted not as easily. For example, the lowest MET-expressing tumor samples also showed high VISTA expression, as indicated by the abundance of blue color in this region of the map, but medium-to-high MET-expressing tumors exhibited high VISTA levels as well. For B7-H4, on the other hand, a lot of tumors expressing low levels of its mRNA show at the same time a high MET expression, apart from the tumors expressing MET, the highest of which show a strong B7-H4 expression. For a quantitative approach, we grouped the data into tumor samples with MET expression higher (“high”) or lower (“low”) than the median MET expression and calculated the means of the expression of the checkpoint proteins. As shown in Figure 5B, the means of the “high” group are significantly higher than the means of the “low” group. For B7-H4, though, the mean is significantly lower than the mean of the “low” group. Lastly, we used the same but ungrouped data to perform a Spearman analysis of correlation (Figure 5C). For PD-L1 with a spearman factor of 0.3, a moderate positive correlation with MET expression could be obtained. For VISTA, however, the analysis resulted in no significant correlation. PD-L2 and B7-H2 showed only a weak positive correlation (for both, a factor of approximately 0.1). In contrast, the analysis of B7-H4 resulted in a weak negative correlation with a Spearman factor near −0.1.

Taking into account these results, the analysis of published mRNA sequencing data could confirm the connection between PD-L1 and MET expression and showed that other checkpoint proteins, like PD-L2, show a weak but positive correlation. The results with our cell lines for VISTA and B7-H2, on the other hand, did not correlate in their tendency with the analyzed sequencing data. However, the cell line Detroit 562, which is highly expressing MET and effectively activated upon HGF stimulation, as shown in this and other publications, nicely replicates the overall tendency of a negative correlation between B7-H4 and MET expression in a set of expression data of a wide variety of cancer entities. However, one has to keep in mind that our investigation is limited to HNSCC tumor cell lines. Other cell types from tumors that could express checkpoint ligands, like different immune cell types or stromal cells, were not included. This could affect a comparison with published sequencing data of tumor tissues.

## 3. Discussion

Immune checkpoint inhibitors (ICIs) gained a lot of attention through successes of antagonistic antibodies against the CTLA-4/CD80/CD86 and PD1/PD-L1 checkpoints. But not all patients benefit from treatment, as demonstrated with HNSCC, where response rates are below 20%. Aside from strategies where ICIs are combined with agents targeting different mechanisms, other immune checkpoints come into the spotlight to solve the problem of low responses to standard ICI medication. Namely, this resistance could be based on other checkpoints being active in these tumors. Therefore, in this work, we investigated the expression of alternative immune checkpoint ligands in HNSCC tumor cells. As we already showed the influence of HGF stimulation on PD-L1 expression, we wanted to investigate if other checkpoint ligands are controlled by this growth factor too. Knowing which pathways are regulating these alternative immune checkpoints is of great importance for finding new treatment options.

PD-L2, like PD-L1, is reported to bind to PD-1 expressed on T and B cells, T-Killer cells, activated monocytes and dendritic cells. Thereby, signaling pathways in these cells are induced that prevent their activation. Compared to PD-L1, the binding between PD-L2 and PD-1 was found to be stronger [22]. Furthermore, its expression could have relevance for anti-PD-1 therapy, because the response of patients positive for both PD-L2 and PD-L1 was stronger. PD-L2 expression was associated with higher overall survival in treated patients as well [23]. PD-L2 is expressed in the majority of patients with HNSCC and was found to be a predictor of poor survival. High PD-L2 expression is correlated to lymph node metastasis in HNSCC [24]. Indeed, we could detect PD-L2 in all of our investigated HNSCC cell lines, although expression was lower than for PD-L1. It could be of benefit to include PD-L2 staining in the scoring for ICIs with PD-1 as point of attack and to consider the results in the assessment which form of ICI, anti-PD1 or anti-PD-L1 is administered. Interestingly, we could show that active HGF/MET signaling has a positive effect on PD-L2 expression, comparable to what we discovered for PD-L1 [19]. Also, for the PAN Cancer data sets, there is a positive correlation between MET expression and the expression of both checkpoint ligands, implying that the activity of HGF/MET signaling in tumors might be of importance for the outcome of PD-1-checkpoint-specific ICI treatment.

The checkpoint protein VISTA not only acts as a ligand but can also act as a receptor on T cells. Apart from immunosuppressive functions, there are several reports showing an activating function. Although it is expressed by a variety of different immune cell types, for several cancer entities an expression on tumor cells could be confirmed [25]. In accordance with this finding, we could detect VISTA not only at the RNA level, but also at the protein level and on the cell surface of most of our tested HNSCC cell lines. In other investigations, VISTA was found to be overexpressed in OSCC tissue samples compared to normal mucosa, and expression positively correlated with lymph node status and the amount of immunosuppression. In combination with low CD8 expression, high VISTA expression was also a predictor for poor overall survival [26]. Furthermore, responders to ICI treatment (mainly anti PD-1) showed lower VISTA levels than non-responders [27]. Consistent with these findings, in a squamous cell carcinoma model, anti-VISTA therapy induced CD8 T-cell activation and, in combination with anti-CTLA4 treatment, led to a stronger reduction in tumor volume [28]. In accordance with the results of the analysis of the PAN Cancer data sets, we could not detect an effect of HGF stimulation on the expression of the protein in the tested HNSCC cancer cell lines.

B7-H2 or ICOSLG is another checkpoint ligand that is not only expressed on antigen-presenting immune cells but also on tumor cells and other non-lymphoid cells [29]. In contrast to the other checkpoint ligands in this study, interaction with its receptor ICOS on effector T cells leads to their differentiation and proliferation. The interaction is, amongst others, important for Th1 and Th2 immunity in response to various infections [30]. In melanoma, ICOS was found to be higher expressed in metastatic lesions of patients showing long survival [31]. On the other hand, B7-H2 expression on melanoma cells was also associated with the activation of regulatory T cells, and hence immune evasion [32]. A study on HNSCC showed that higher TNM stages correlated positively with B7-H2 expression on tumor cells. A higher risk of lymph node metastasis was observed as well. Furthermore, patients showing a higher B7-H2 expression on tumor cells had shorter overall survival. B7-H2 expression correlated with a high FoxP3 expression in TILs, an indicator for regulatory T cells and an immunosuppressive tumor environment [33]. In the HNSCC cell lines we tested, we could detect only small levels of mRNA, and on the cell surface, only weak staining. We could not confirm in our cell lines the tendency for higher expression of this protein when there are also high MET levels present in whole TCGA data. In fact, if affected at all, expression was reduced with HGF stimulation.

B7-H4 inhibits T-cell proliferation and cytokine production and was reported to be associated with immunosuppression in several cancer entities, e.g., colorectal cancer, lung cancer and breast cancer. For HNSCC, there were contradictory results obtained, ranging from high expression and being an indicator for poor prognosis in OSCC [34] to mainly low expression without significant correlation in a study comprising several HNSCC locations [35]. Investigations of B7-H4 function in vitro are hampered by the fact that the protein is unstably present on the cell surface and often only detected there on fresh tumor samples [20,21]. Interestingly, the expression of B7-H4 is often reported to be positively correlated with epithelial–mesenchymal transition. In breast cancer, the opposite was reported: downregulation of the protein increased cell proliferation, migration and invasion [36]. HGF/MET signaling is crucial for EMT processes in development and cancer [37,38]. Mesenchymal markers are induced upon MET receptor activation. On the other hand, in breast cancer, B7-H4 has been found to be negatively correlated with mesenchymal markers and positively correlated with epithelial markers [39]. In another investigation, knockout of B7-H4 in breast cancer tumor cells prompted EMT and a higher expression of mesenchymal markers [36]. A negative connection between HGF/MET signaling and B7-H4 is therefore plausible. The effect of HGF/MET signaling on B7-H4 and the role of B7-H4 downregulation in EMT should therefore be investigated further.

## 4. Materials and Methods

### 4.1. Cell Lines

Cell lines were purchased from ATCC (Detroit 562, FaDu and SCC-9, Manassas, VA, USA) and DSMZ (BHY, HN, SCC-154, Braunschweig, Germany). Cells were cultivated as described in [40].

### 4.2. Western Blot

Cell suspensions of 2 mL were seeded in 6-well plates at a density of 400,000 cells/mL and on the next day were treated with HGF (50 ng/mL), HGF and Foretinib (0.5 µM), Foretinib alone or remained untreated. After 6 h or 24 h of incubation, cell lysates and Western blot samples were prepared as described elsewhere [40]. HGF was obtained from Thermo Fisher Scientific (PHG0254, Waltham, MA, USA) or Abcam (Ab259401, Cambridge, Great Britain). Foretinib was purchased from Selleck Chemicals (Houston, TX, USA). SDS-PAGE was run, and gels blotted on PVDF membranes were blocked for one hour with 5% (*w*/*v*) dry milk in TBS. Incubation with the following primary antibodies was performed over night at 4 °C on a roller according to the instructions provided by the manufacturers: anti-B7-H4 (D1M8I), anit-PD-L2 (D7U8C), anti-VISTA (D1L2G, all Cell Signaling, Danvers, MA, USA) and anti-ICOS-LG (EPR6071, Thermo Fisher Scientific, Waltham, MA, USA). Membranes were washed three times for 5 min with TBS-0.05% Tween, incubated for one hour with the corresponding secondary antibody (anti-rabbit, HRP-coupled, Cell signaling, Danvers, MA, USA), and further processed to signal detection, as described elsewhere [40]. Western blot bands were quantified using the software Image Lab 6.0.1 (Bio-Rad, Hercules, CA, USA). Band intensities of the proteins of interest were adjusted using the relative intensities of the corresponding Vinculin control band on the same blot. For representation, intensities were normalized to the intensities of the respective untreated sample.

### 4.3. Flow Cytometry

First, 1 Mio of cells was seeded in 6-well-plates, stimulated as described above (Section 4.2, Western blot section) and stained for flow cytometry after 24 h of incubation. Cells were detached from the plate using Trypsin-EDTA (Biochrom, Merck, Darmstadt, Germany), spun down and incubated with the following antibodies at 4 °C diluted in FACS buffer (PBS containing 0.5% BSA and 1 mM EDTA) according to the instructions of the manufacturers: APC-coupled anti-B7-H4 (Thermo Fisher Scientific, Waltham, MA, USA), PE-coupled anti-PD-L2 (130-098-733, Miltenyi Biotec, Bergisch-Gladbach, Germany), PE-coupled anti-VISTA (B7H5DS8, Thermo Fisher Scientific, Waltham, MA, USA) and APC-coupled anti-ICOS-LG (130-098-531, Miltenyi Biotec). Cells were spun down, washed two times with FACS-Buffer and measured in a flow cytometer (Attune Nxt, Thermo Fisher Scientific, Waltham, MA, USA).

### 4.4. Immunofluorescence

An amount of 20.000 cells per well was seeded in black 96-well plates with transparent bottoms (Greiner, Frickenhausen, Germany), stimulated on the next day with HGF or a combination of HGF and Foretinib (see Section 4.2, Western blot for details) and stained 24 h later. Untreated cells were used as a control. For staining, cells were washed with PBS, fixed for 15 min with 4% Formaldehyde in PBS, washed again and treated with 0.3% Triton^TM^X-100 for permeabilization for 15 min. For blocking, cells were incubated for 1 h with a 5% dilution of goat serum (#5425, Cell signaling) in PBS containing 0.3% TritonTMX-100. Incubation with an antibody specific for B7-H4 (1:90, 1788, Neobiotechnology, Union City, CA, USA) or PD-L1 (1:200, #86744, Cell Signaling, Danvers, MA, USA) was conducted overnight at 4 °C in PBS, 0.5% BSA and 0.3% TritonTMX-100, followed by three washing steps and an incubation with an Alexa Fluor 488-coupled secondary antibody (1:1000, #4412 or #4408, Cell Signaling, Danvers, MA, USA) and Hoechst 33342 (1:1000, Thermo Fisher Scientific, Waltham, MA, USA) for one hour on the next day. After three additional washing steps, stained cells were analyzed in an automated cell analyzing system using the cell scoring analysis program (Image Express Pico, Molecular Devices, San José, CA, USA). Cells identified via their stained nuclei were scored as positive for the given protein if staining exceeded a given background intensity. Cells stained with secondary antibody and Hoechst 33342 only were used to determine background intensity.

### 4.5. Data Analysis

The mRNA sequencing raw data used in Figure 1 can be found at the Gene Expression Omnibus under the accession number GSE135552. A detailed protocol of the sample preparation, mRNA sequencing, data processing and additional analysis of the data can be found elsewhere [17]. Heatmaps were prepared using Heatmapper (www.heatmapper.ca, accessed on 27 October 2023) [41].

mRNA sequencing data of the TCGA PAN Cancer project (https://www.cancer.gov.tcga, accessed on 27 October 2023) were arranged using the XENA platform (https://xenabrowser.net/, accessed on 27 October 2023) [42]. Data of normal tissue (normal tissue solid) were excluded, and remaining samples were divided into two groups, one “low” group with samples showing a normalized expression of MET lower than the median of 10.7 and one “high” group with samples showing a normalized expression higher than the median. Data were then plotted and statistically analyzed using Graph Pad Prism.

## Data Availability

The raw data supporting the conclusions of this article will be made available by the authors on request.

## Supplementary Materials

The following supporting information can be downloaded at https://www.mdpi.com/article/10.3390/ijms25137334/s1.
