# Supplementary figures and images for: Differential Immune Checkpoint Protein Expression in HNSCC: The Role of HGF/MET Signaling
Source: Int J Mol Sci. 2024 Jul 4;25(13):7334. doi: 10.3390/ijms25137334 (PMC11242282; doi:10.3390/ijms25137334)

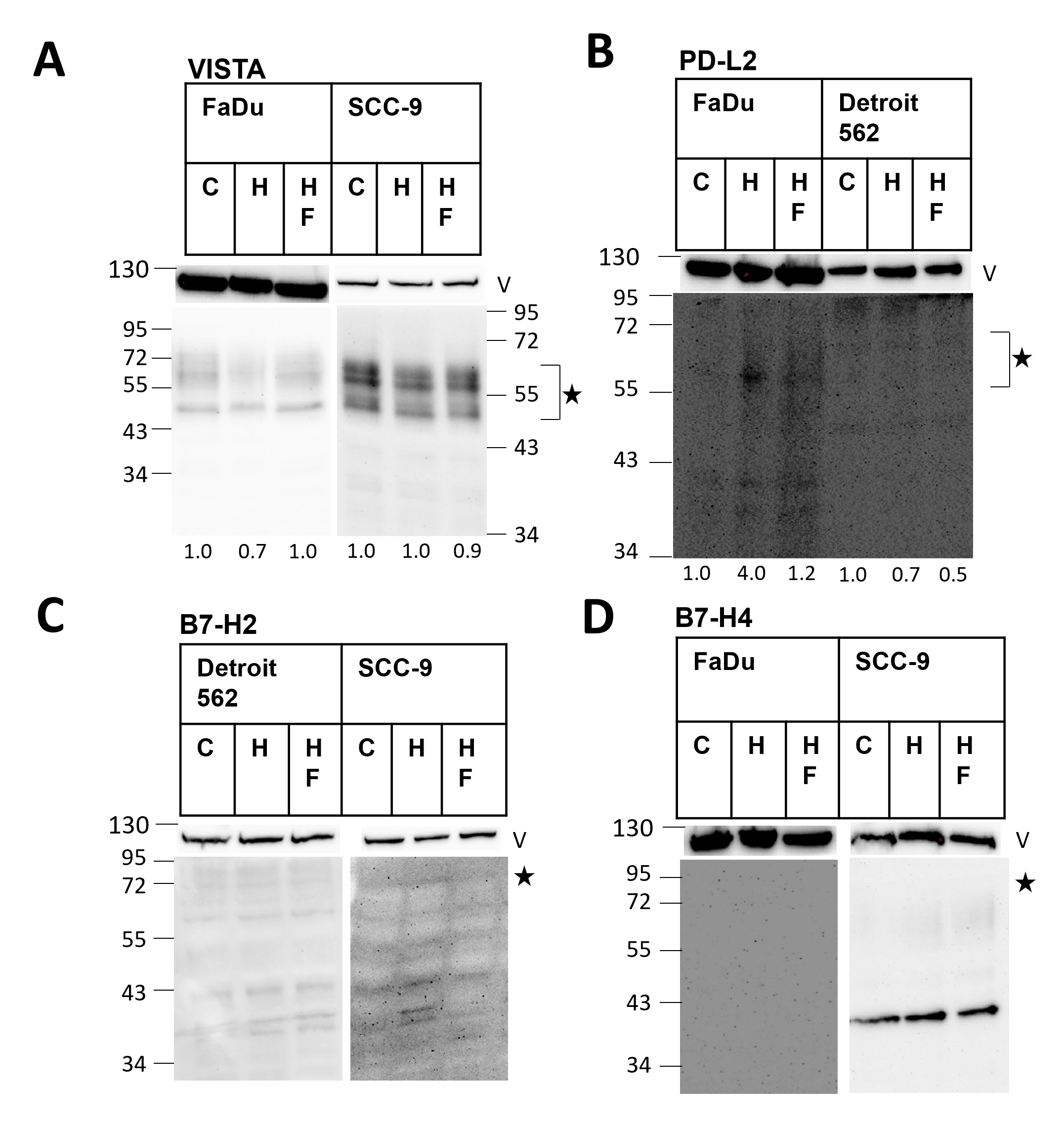

Supplement: Supplementary file 1 [file ijms-25-07334-s001.zip › ijms-3040261-supplementary conversion/Supplementary data/Figure S1.tif]

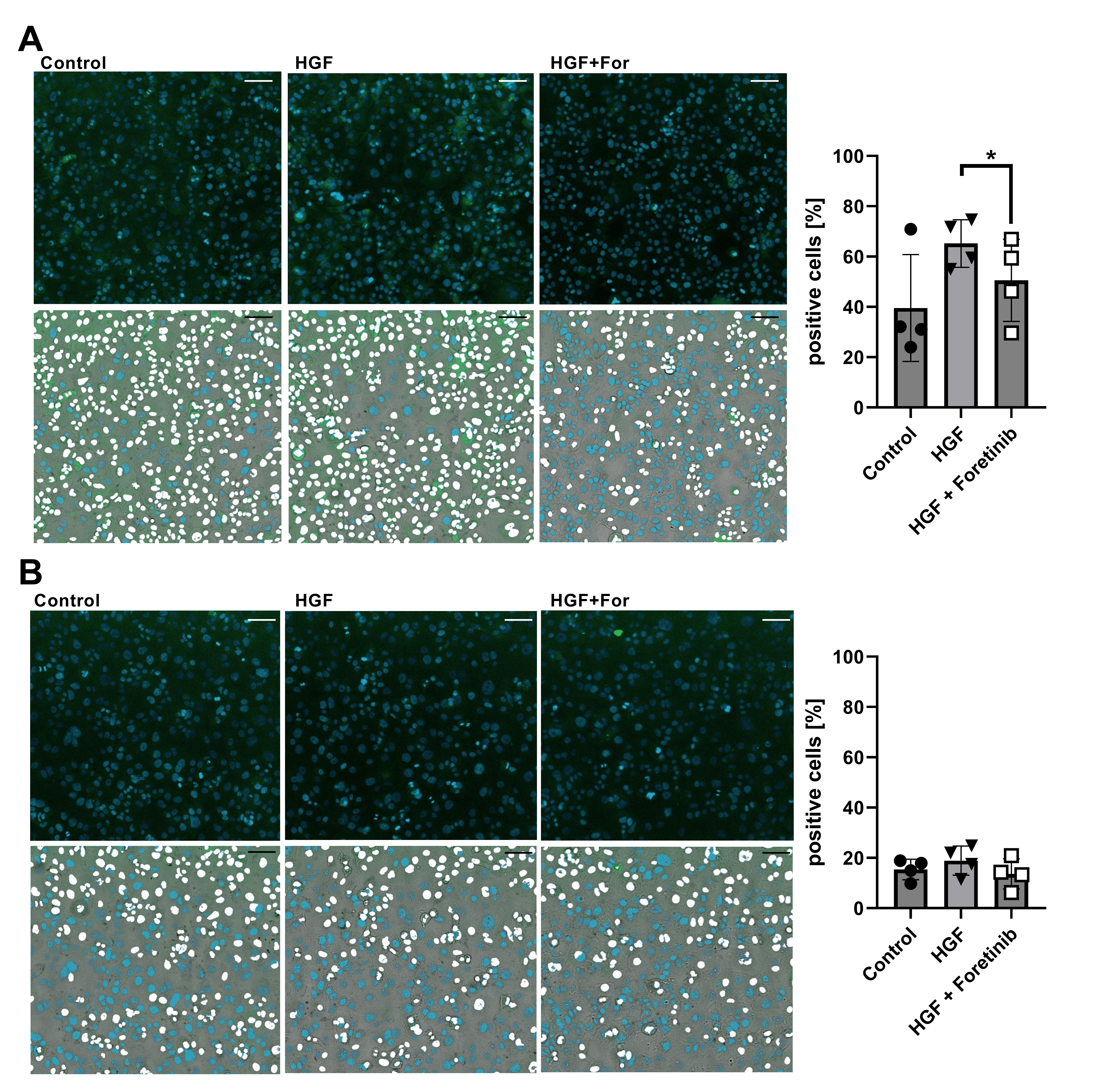

Supplement: Supplementary file 1 [file ijms-25-07334-s001.zip › ijms-3040261-supplementary conversion/Supplementary data/Figure S2 revised.tif]
